# Can transesophageal echocardiography be safely deferred? A low-cost predictive model using D-dimer to exclude left atrial thrombus in treatment-naïve rheumatic mitral stenosis

**DOI:** 10.3389/fcvm.2026.1780267

**Published:** 2026-08-10

**Authors:** Nawar A. Al-Wather, Mohammed Al-Kebsi, Abdulhafeedh Al-Habeet, Ahmed Al-Motarreb, Hesham Al-Fakih

**Affiliations:** 1 Department of Cardiology, Faculty of Medicine and Health Sciences, Sana'a University, Sana'a, Yemen; 2 Department of Epidemiology and Biostatistics, Faculty of Medical Sciences, Al-Razi University, Sana’a, Yemen

**Keywords:** atrial fibrillation, D-dimer, diagnostic accuracy, left atrial thrombus, rheumatic severe mitral stenosis

## Abstract

**Background:**

Transesophageal echocardiography (TEE) is the reference standard for excluding left atrial thrombus (LAT) in rheumatic severe mitral stenosis (SMS), yet its availability is limited in many low-resource settings. A reliable, low-cost strategy to safely defer TEE would have a significant clinical impact. This study evaluated whether D-dimer–based predictive models can accurately exclude newly detected LAT in treatment-naïve patients with rheumatic SMS.

**Methods:**

Sixty-eight consecutive patients with rheumatic SMS who were not receiving anticoagulation underwent D-dimer testing, transthoracic echocardiography, and TEE. Associations with LAT were assessed using conventional and Firth-penalized logistic regression. Diagnostic performance was evaluated using receiver operating characteristic analysis with 1,000-bootstrap internal validation. Two composite models were examined: D-dimer combined with atrial fibrillation (AF) and D-dimer combined with mitral valve area (MVA).

**Results:**

LAT was newly detected in 12 patients (17.6%). D-dimer levels were significantly higher in LAT-positive patients (943.5 vs. 168.5 ng/mL, *p* < 0.001). The optimal D-dimer cut-off (360.6 ng/mL) achieved 100% sensitivity, 71.4% specificity, and an optimism-corrected AUC of 0.935, outperforming the conventional 500 ng/mL threshold (sensitivity 75%). In adjusted models, D-dimer remained a strong independent predictor of LAT (per SD: AOR 6.91; 95% CI 2.72–17.54). Composite models demonstrated similarly high discrimination (AUC 0.933–0.940); adding AF did not improve performance, whereas incorporating MVA increased specificity to 100%.

**Conclusions:**

D-dimer may help exclude LAT in treatment-naïve patients with rheumatic SMS. Its potential role in supporting more selective TEE use in resource-limited settings warrants further evaluation in larger prospective multicenter studies.

## 1 Introduction

Rheumatic heart disease (RHD) remains a major cause of valvular heart disease in low- and middle-income countries, where limited preventive care and diagnostic infrastructure continue to drive high morbidity and mortality (1–3). Among its sequelae, rheumatic severe mitral stenosis (SMS) accounts for a substantial disease burden, particularly in younger populations. Patients with SMS are at increased risk of left atrial thrombus (LAT) due to progressive atrial dilation and blood stasis—pathophysiologic conditions that predispose to systemic embolization and complicate interventions such as percutaneous balloon mitral valvotomy (PBMV) (4, 5). Prompt identification of LAT is therefore essential to prevent embolic events and ensure procedural safety.

Transesophageal echocardiography (TEE) remains the diagnostic gold standard for left atrial thrombus (LAT) detection; however, its high cost, invasiveness, and limited availability significantly restrict its routine use in resource-constrained healthcare systems (6, 7), such as those in Yemen. In addition to concerns related to patient discomfort and procedural safety, access to TEE is severely limited nationwide, with the procedure available in only two specialized centers. As a result, waiting times for TEE frequently exceed two weeks, during which patients remain exposed to ongoing thromboembolic risk and potential clinical deterioration. Although transthoracic echocardiography (TTE) can identify a proportion of left atrial thrombi, its sensitivity, particularly for left atrial appendage (LAA) thrombi, remains limited. In such environments, reliance on TEE as the sole confirmatory modality may delay diagnosis, restrict timely access to percutaneous balloon mitral valvotomy (PBMV), and increase both patient risk and healthcare expenditure. These systemic constraints underscore the urgent need for simple, inexpensive, and non-invasive diagnostic strategies that can reliably exclude LAT before intervention.

D-dimer, a fibrin degradation product reflecting ongoing thrombogenesis and fibrinolysis, is well established as a rule-out biomarker for venous thromboembolism (VTE) (8, 9). Beyond its diagnostic performance, D-dimer testing offers practical advantages in terms of safety, rapid turnaround time, and ease of implementation. Its potential role in detecting LAT among patients with rheumatic mitral stenosis (RMS) is pathophysiologically plausible yet remains underexplored, particularly in low-income settings where access to TEE is limited. While D-dimer testing may not be universally available in all low-resource environments, it is frequently accessible in many secondary and tertiary care centers and is substantially less resource-intensive than TEE. Importantly, its clinical utility lies not in replacing echocardiography but in serving as an adjunctive rule-out tool that may optimize patient selection for advanced imaging.

Most existing studies evaluating D-dimer in this context have focused on anticoagulated or atrial fibrillation (AF)–restricted populations (10–12), thereby limiting generalizability and obscuring the intrinsic relationship between D-dimer levels and active thrombus formation. The present study addresses these limitations by evaluating newly diagnosed LAT in treatment-naïve patients, encompassing both AF and non-AF subgroups. This design enables a more comprehensive and unconfounded assessment of the biological association between D-dimer and LAT in rheumatic SMS.

From a public health perspective, this investigation holds particular relevance for resource-limited countries such as Yemen, where RHD remains endemic (13) and advanced imaging facilities are scarce. Establishing D-dimer as a simple, low-cost, and reliable rule-out biomarker could substantially reduce unnecessary reliance on TEE, facilitate earlier risk stratification, and improve access to timely intervention. Accordingly, this study was designed to evaluate the diagnostic and predictive performance of D-dimer—both alone and in composite models incorporating AF and mitral valve area (MVA)—for excluding newly detected LAT in treatment-naïve patients with rheumatic SMS, thereby providing a pragmatic and context-appropriate diagnostic framework for rheumatic populations in low-resource settings and offering an unbiased assessment of D-dimer's true diagnostic value.

## 2 Patients and methods

### 2.1 Study design and population

Between June 2024 and July 2025, consecutive adult patients diagnosed with rheumatic SMS who were scheduled for TEE before percutaneous transvenous mitral commissurotomy (PTMC) at Al-Thawra Modern General Hospital (TMGH), Sana'a, Yemen, were prospectively enrolled.

Patients previously diagnosed with LAT or those who had received any form of anticoagulant therapy (oral or parenteral) within the preceding three weeks were excluded. Additional exclusion criteria included the presence of systemic inflammatory or malignant diseases, acute infections, renal failure, recent surgery, trauma, or thrombotic events (deep vein thrombosis, pulmonary embolism, or stroke); moderate to severe mitral regurgitation; moderate to severe aortic stenosis or regurgitation; and pregnancy.

Due to the specific clinical and geographical context of our tertiary referral center, a formal *a priori* sample size calculation was not performed. Instead, the sample size was determined by the maximum number of eligible consecutive patients presenting during the predefined 14-month study period. This time-bound consecutive recruitment strategy was adopted to minimize selection bias and to ensure that the study cohort adequately reflected the target population of patients with severe, treatment-naïve RMS encountered in routine clinical practice.

All participants provided written informed consent before enrollment. The study protocol was reviewed and approved by the Ethics Review Committee of TMGH and conducted in accordance with the principles outlined in the Declaration of Helsinki (revised in October 2013). Patients were not deprived of any standard medical care, and all received appropriate treatment and medications as clinically indicated, including anticoagulation therapy when necessary.

### 2.2 Data collection

#### 2.2.1 Baseline clinical and echocardiographic characteristics

Baseline demographic and clinical data were prospectively collected through direct patient interviews and standardized case report forms at the time of enrollment. Variables included age, gender, cardiac rhythm (sinus rhythm vs. AF), NYHA functional class, prior history of PTMC, and major comorbidities such as diabetes mellitus (DM), hypertension (HTN), and previous thromboembolic events. Echocardiographic parameters reflecting mitral stenosis severity were obtained using TTE, including MVA, mean transmitral gradient, pulmonary artery systolic pressure (PASP), and left atrial (LA) size. Additional findings, such as involvement of other cardiac valves, left ventricular (LV) dimensions, and LV ejection fraction (LVEF), were also systematically assessed.

#### 2.2.2 Transesophageal echocardiography

Patients referred to the TEE unit of TMGH Cardiac Center before PTMC were prepared by receiving a full explanation of the procedure. TEE was then performed under local pharyngeal anesthesia using lidocaine spray by two experienced echocardiographers who were blinded to the D-dimer results.

All examinations were carried out using a Vivid 7 ultrasound system (GE HealthCare, USA) equipped with a multiplane 2D TEE probe. Each patient was positioned in the left lateral decubitus position, and the probe was gently introduced through an oral mouthpiece. Standard mid-esophageal imaging planes were obtained. To ensure comprehensive visualization of the LAA and definitively exclude localized thrombi, the LAA was visualized in at least multiple planes, including a 0°, 45°, 90°, and 135° multiplanar angle. This systematic multi-angle interrogation permitted a complete evaluation of the LAA ostium, body, and lobes.

A thrombus was defined as a discrete, homogeneous or heterogeneous echogenic mass distinct from the endocardial surface, with or without central echolucency, typically displaying irregular margins. Such a mass located within the LAA and possibly protruding into the LA cavity was considered diagnostic of LAT.

#### 2.2.3 Echocardiographic definitions

Rheumatic SMS was defined as a MVA ≤ 1.5 cm^2^, measured by planimetry in the parasternal short-axis view and corroborated, when feasible, by the pressure half-time method (14).

LAT was identified as a discrete, echogenic mass, either homogeneous or heterogeneous, clearly distinct from the endocardial surface of the LA or LAA (15), and visualized in at least two imaging planes.

Spontaneous echo contrast (SEC) was characterized by dynamic, smoke-like echogenicity within the LA or LAA, displaying a swirling pattern at low flow velocities suggestive of blood stasis and increased erythrocyte aggregation (16).

#### 2.2.4 Blood sampling and D-dimer analysis

Venous blood samples (1.8 mL) were collected from each patient within three hours of completing the TEE procedure to ensure temporal alignment with imaging findings. Samples were drawn into 3.2% sodium citrate Vacutainer tubes, gently inverted several times to ensure adequate mixing, and then centrifuged at 1,500 g for 15 min at room temperature (18–24 °C) to obtain platelet-poor plasma.

The supernatant plasma was analyzed using a fully automated Mindray BS-240 Pro biochemistry analyzer (Mindray, Shenzhen, China) with a latex-enhanced immunoturbidimetric assay for quantitative D-dimer determination. When immediate testing was not feasible, samples were flash-frozen at −70 °C and later thawed at 37 °C for three minutes before analysis. D-dimer concentrations were expressed in fibrinogen equivalent units (FEU, ng/mL), with a validated analytical precision suitable for modeling and rule-out threshold determination around 500 ng/mL FEU.

### 2.3 Statistical analyses

All statistical analyses were performed using R software (version 4.2.0; R Foundation for Statistical Computing, Vienna, Austria). Reporting of the predictive modeling analyses was guided by the Transparent Reporting of a multivariable prediction model for Individual Prognosis Or Diagnosis (TRIPOD) recommendations (17). Continuous variables were assessed for normality using the Shapiro–Wilk test and summarized as mean ± standard deviation (SD) or median [interquartile range (IQR)], as appropriate. Categorical variables were expressed as frequencies and percentages. Between-group differences were examined using the Student's t-test or Mann–Whitney U test for continuous data and the chi-square or Fisher's exact test for categorical data.

Because the number of LAT events was limited (*n* = 12), model development adhered to a parsimonious strategy guided by the events-per-variable principle. To minimize the risk of overfitting, a full multivariable analysis was deliberately avoided; instead, composite models were strictly restricted to include only one additional variable alongside D-dimer. Furthermore, continuous predictors, specifically D-dimer and MVA, were entered as linear terms on the logit scale. Although non-linear relationships cannot be excluded, the limited sample size and low event count constrained the use of more flexible approaches such as restricted cubic splines, which would have consumed additional degrees of freedom and increased the risk of model overfitting and unstable parameter estimates.

Initially, a purely data-driven variable selection was performed using the least absolute shrinkage and selection operator (LASSO) regression, employing cross-validation to determine the optimal tuning parameter (lambda). The LASSO penalty shrank the coefficients of all other candidate variables to zero, identifying D-dimer as the sole independent predictor retained across stability thresholds. In the unadjusted analysis, D-dimer level was then evaluated as the primary predictor of LAT using standard logistic regression.

To address the limited number of events and potential statistical separation issues, Firth's penalized likelihood logistic regression was subsequently applied to refine the D-dimer–only model and to construct two additional restricted composite models (incorporating either AF or MVA as a single covariate alongside D-dimer). While not selected by the data-driven LASSO algorithm, AF and MVA were introduced in a theory-driven manner due to their established clinical and pathophysiological relevance as determinants of blood stasis, thrombogenesis (Virchow's triad), and hemodynamic severity in RMS. This enabled us to evaluate their incremental predictive value over the biomarker alone. Adjusted odds ratios (AORs) with 95% confidence intervals (CIs) were computed accordingly.

Model discrimination was quantified by the area under the receiver operating characteristic curve (AUC) with 95% CIs obtained through 2,000 bootstrap resamples. The optimal probability threshold was determined using Youden's J index, balancing sensitivity and specificity. 95% CIs for diagnostic performance metrics (sensitivity, specificity, and predictive values) were calculated using the exact binomial (Clopper-Pearson) method. Predicted probabilities of LAT were computed from a Firth logistic model using the logistic (inverse-logit) transformation. These probabilities were used to generate ROC curves, calibration plots, and predicted probability curves to visualize the relationship between D-dimer levels and the model-predicted risk of LAT. The predicted probability curves included 95% confidence bands, with the Youden threshold and the conventional clinical cutoff (500 ng/mL) explicitly marked for clinical interpretability.

Calibration was evaluated by plotting observed versus predicted probabilities using a locally weighted scatterplot smoothing calibration curve, supported by the Brier score and Hosmer–Lemeshow goodness-of-fit test. Internal validation was performed using 1,000 bootstrap resamples, providing optimism-corrected estimates of the AUC and calibration slope in accordance with the methodological recommendations of Harrell and Steyerberg for small-sample prediction modeling (18, 19). All tests were two-tailed, and *p* < 0.05 was considered statistically significant.

## 3 Results

A total of 170 consecutive patients with rheumatic SMS were screened. After excluding 102 ineligible cases (35 on warfarin, 25 with infection, 17 with mixed lesions, 15 pregnant, and 10 with renal impairment), 68 treatment-naïve patients were analyzed. LAT was identified in 12 (17.6%) and SEC in 16 (23.5%).

### 3.1 Baseline clinical and echocardiographic characteristics

Table 1 summarizes the clinical and echocardiographic characteristics according to LAT status. Patients with LAT tended to be younger, though the age difference was not statistically significant (*p* = 0.076). There were no differences between the two groups regarding gender, smoking, khat chewing, or comorbidities such as HTN or DM. Conversely, AF was significantly more prevalent among patients with LAT (41.7% vs. 9.0%, *p* < 0.001).

**Table 1:** Baseline clinical and echocardiographic characteristics of patients with and without left atrial thrombus (LAT) in rheumatic severe mitral stenosis (SMS).

Variables	LAT present (*N* = 12)	LAT absent (*N* = 56)	*P*
Age Mean ± SD	32.3 ± 10.5	38.4 ± 10.5	0.076
Female gender, *n* (%)	3 (25.0)	8 (14.3)	0.298
Smoker, *n* (%)	1 (8.3)	2 (3.6)	0.447
Khat chewer, *n* (%)	2 (16.7)	4 (7.1)	0.285
DM, *n* (%)	1 (8.3)	0 (0.0)	0.176
HTN, *n* (%)	1 (8.3)	1 (1.8)	0.324
History of BMV, *n* (%)	0 (0.0)	4 (7.1)	0.451
NYHA classification, *n* (%)			
I	2 (16.7)	17 (30.4)	0.130
II	5 (41.7)	30 (53.6)	
II	5 (41.7)	9 (16.1)	
AF, *n* (%)	7 (58.3)	5 (8.9)	<0.001
LA diameter (mm), median (IQR)	59.0 (56.25, 61.0)	52.5 (46.25, 56.0)	<0.001
LVEF (%), mean ± SD	64.2 ± 4.9	62.6 ± 4.7	0.301
MVA (cm^2^), median	0.85 (0.7, 0.97)	1.0 (0.9, 1.2)	0.008
MVG (mmHg), median (IQR)	12.5 (8.0, 16.75)	9.0 (7.0, 11.0)	0.002
PASP (mmHg), median (IQR)	69.0 (61.2, 89.7)	60.8 (48.5, 75.0)	0.011
ESD, median (IQR)	28.0 (23.0, 31.7)	27.5 (25.0, 30.7)	0.588
EDD, median (IQR)	43.5 (39.2, 47.7)	46.0 (44.0, 48.0)	0.083
D-Dimer result	943.5 (550.0, 987.7)	168.5 (139.0, 433.5)	<0.001

AF, atrial fibrillation; BMV, balloon mitral valvotomy; DM, diabetes mellitus; EDD, end-diastolic diameter; ESD, end-systolic diameter; HTN, hypertension; IRQ, interquartile range; LA, left atrial; LAT, left atrial thrombus; MVA, mitral valve area; MVG, mean transvalvular gradients; PASP, pulmonary artery systolic pressure; SD, standard deviation.

Echocardiographically, LAT-positive patients showed more advanced structural and hemodynamic alterations, including significantly larger LA diameters (59.0 vs. 52.5 mm, *p* < 0.001), smaller MVA (0.85 vs. 1.0 cm^2^, *p* = 0.008), and higher mean transvalvular gradient (MVG) (12.5 vs. 9.0 mmHg, *p* = 0.002). PASP was also elevated in the LAT group (69.0 vs. 60.8 mmHg, *p* = 0.011), indicating greater pulmonary HTN and hemodynamic burden. Importantly, the median D-dimer level was markedly higher in patients with LAT compared with those without (943.5 vs. 168.5 ng/mL, *p* < 0.001).

### 3.2 Univariate and multivariable regression

As presented in Table 2, univariate logistic regression demonstrated a strong association between D-dimer level and LAT (per SD increase: OR 8.39, 95% CI 2.92–24.11; *p* < 0.001). Firth's penalized logistic regression confirmed the robustness of this relationship. In the D-dimer–only model, each SD increase corresponded to an AOR of 6.91 (95% CI 2.72–17.54; *p* < 0.001). Composite models yielded comparable effect sizes, including D-dimer+AF (AOR 5.17; 95% CI 2.40–11.12; *p* < 0.001) and D-dimer+MVA (AOR 5.43; 95% CI 2.48–11.92; *p* < 0.001).

**Table 2:** Univariate and firth's penalized logistic regression analyses evaluating D-dimer (alone and in composite models) for prediction of LAT in rheumatic SMS patients.

Model	Increment Type	*β* (SE)	OR (95% CI)	*P*
Univariate logistic regression				
D-dimer–only	Per 1 unit increase	0.0068 (±0.0017)	1.007 (1.003–1.010)	< 0.001
	Per SD increase		8.393 (2.925–24.116)	
Firth's penalized logistic regression				
D-dimer–only	Per 1 unit increase	0.0062 (± 0.0015)	1.006 (1.004–1.010)	< 0.001
	Per SD increase		6.914 (2.721–17.540)	
D-dimer+AF	Per 1 unit increase	0.060 (± 0.014)	1.067 (1.030–1.091)	< 0.001
	Per SD increase		5.172 (2.401–11.124)	
D-dimer+MVA	Per 1 unit increase	0.062 (± 0.015)	1.062 (1.035–1.096)	< 0.001
	Per SD increase		5.433 (2.480–11.921)	

AF, atrial fibrillation; CI, confidence interval; MVA, mitral valve area; OR, odds ratio; SD, standard deviation; SE, standard error.

### 3.3 Model discrimination, calibration, and internal validation

**Figure 1:**
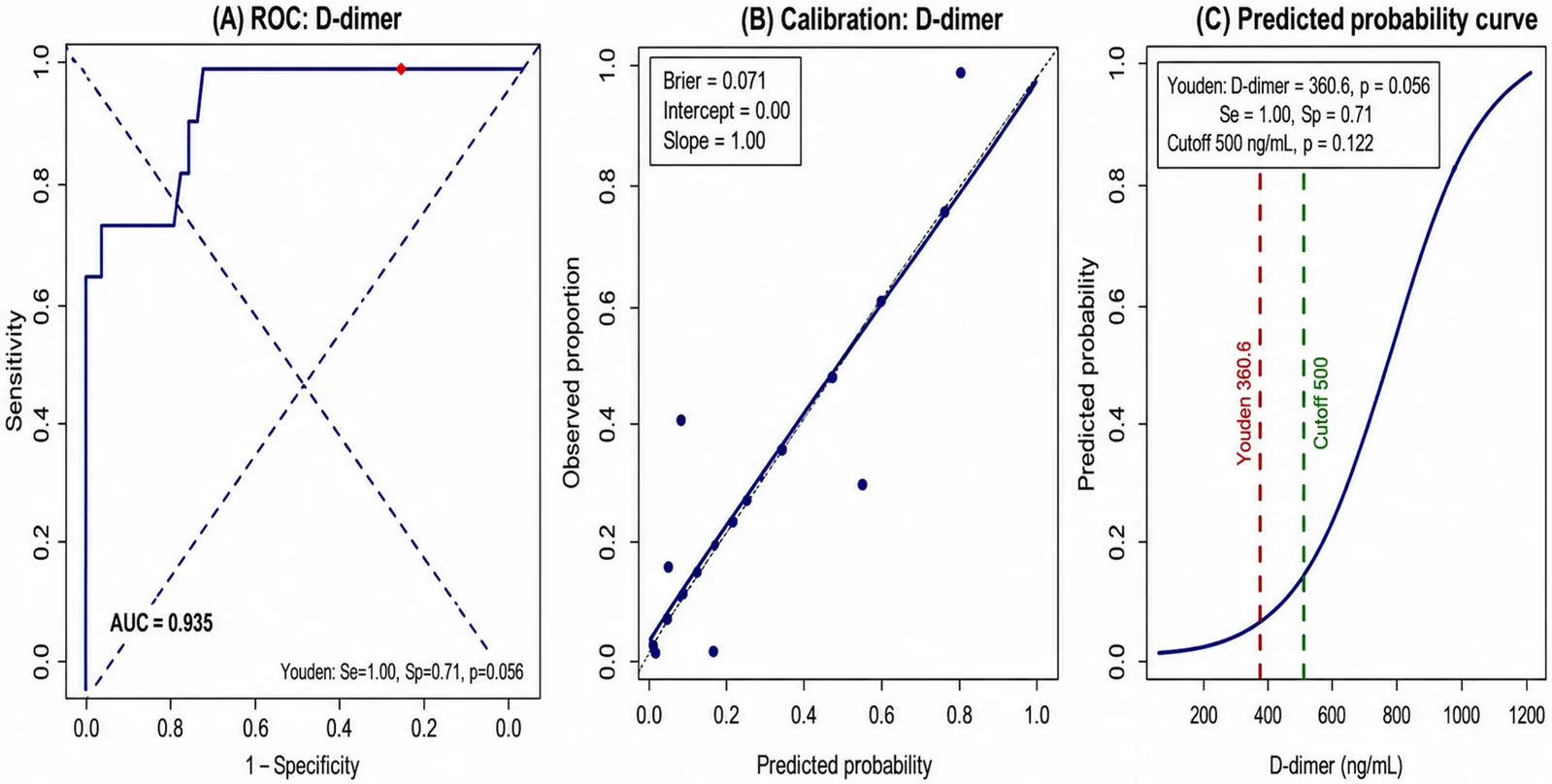
Discrimination **(A)**, calibration **(B)**, and predicted probability curve **(C)** of the D-dimer model for predicting LAT in rheumatic SMS patients. Panels **(A)** and **(B)** display the ROC and calibration plots, showing excellent discrimination (AUC = 0.935) and strong calibration performance (slope = 1.00, Brier = 0.071). Panel **(C)** illustrates the predicted probability curve, with the red dashed line indicating the Youden threshold (360.6 ng/mL), corresponding respectively to sensitivity = 1.00 and specificity = 0.71, and the green dashed line marking the clinical cutoff (500 ng/mL).

**Figure 2:**
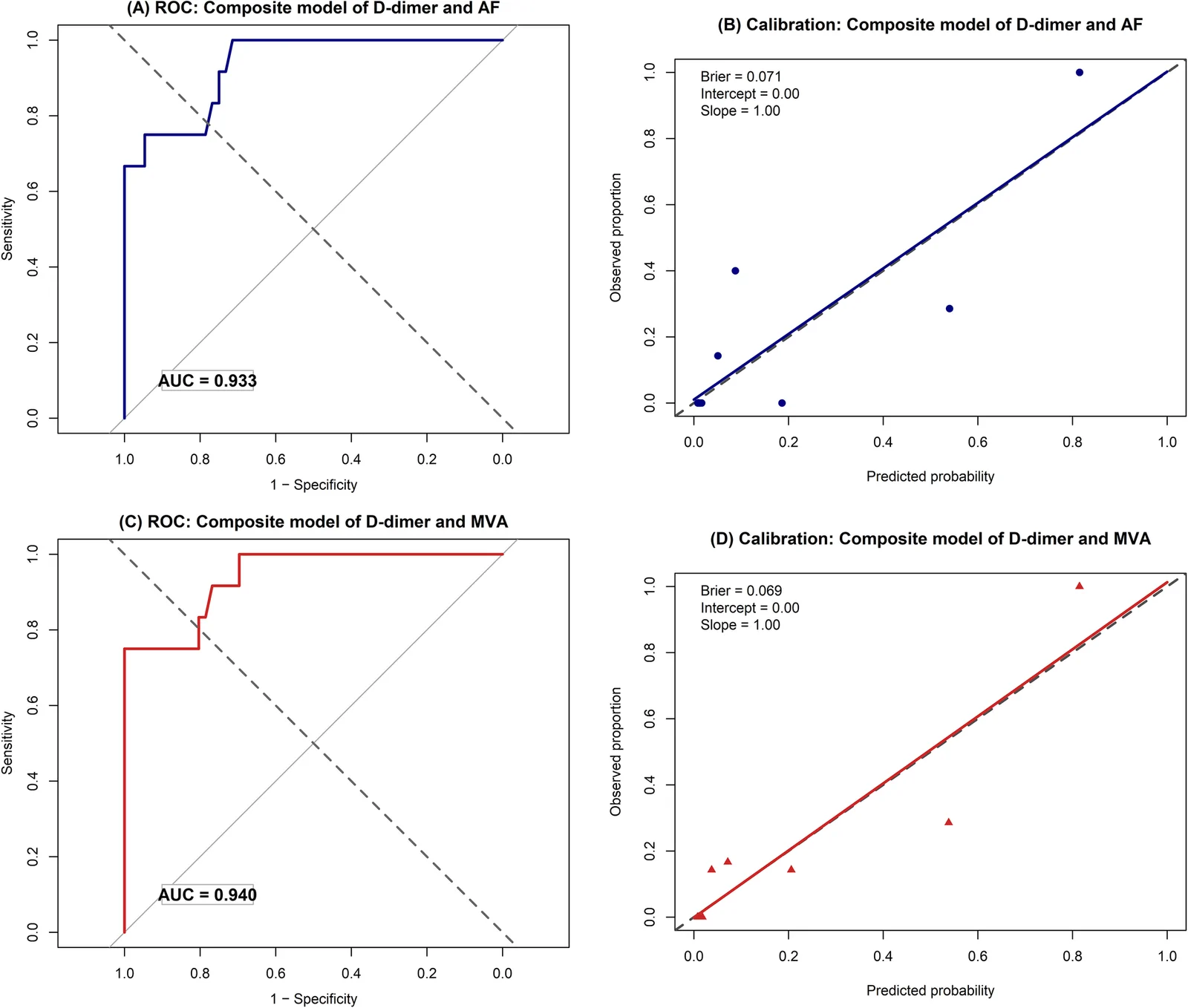
ROC and calibration plots of the composite models for predicting LAT in rheumatic SMS patients. Panels **(A,B)** show the composite model of D-dimer and MVA, and panels **(C,D)** show the composite model of D-dimer and MVA. In panels **(B–D)**, the dashed diagonal line denotes perfect calibration, and the solid line indicates the observed fit.

Performance analyses (Table 3) showed consistently high discrimination across all models, with AUC values of 0.935 for the D-dimer–only model, 0.933 for the D-dimer+AF model, and 0.940 for the D-dimer+MVA model. Calibration slopes were close to 1.0 (ranging from 0.904 to 1.140), and Brier scores remained low (0.070–0.073), indicating strong agreement between predicted and observed risk. Internal validation with 1,000 bootstrap resamples demonstrated minimal optimism in AUC (≤0.002), confirming excellent model stability. ROC curves (Figure 1) showed near-complete overlap between the D-dimer–only and D-dimer+AF models, while the D-dimer+MVA model displayed a marginally higher curve in the high-specificity region. Calibration plots (Figure 2) demonstrated an appropriate fit for all models.

**Table 3:** Discrimination, calibration, and internal validation performance of D-dimer–based models for prediction of LAT in rheumatic SMS patients.

Model	Performance statistics	Apparent performance in the original sample	Internal validation		
			Bootstrap performance (mean of 1,000 resamples)	Optimism corrected performance	Mean optimism (*Δ*)
D-dimer–only	C statistic (AUC)	0.935	0.935	0.935	0.00002
	Calibration	Slope: 1.10 Intercept: 0.021	Slope: 0.9622 Intercept: −0.050	Slope: 0.962 Intercept: −0.06	Slope: 0.165 Intercept: 0.081
	Brier Score	0.0713	0.070	0.070	0.001
	Hosmer–Lemeshow *p*-value	0.165			
D-dimer+AF	C statistic	0.933	0.931	0.931	0.002
	Calibration	Slope: 0.906 Intercept: −0.022	Slope: 1.088 Intercept: −0.129	Slope: 1.088 Intercept: −0.129	Slope: −0.182 Intercept: 0.107
	Brier Score	0.071	0.072	0.072	−0.001
	Hosmer–Lemeshow *p*-value	0.120			
D-dimer+MVA	C statistic	0.940	0.938	0 938	0.002
	Calibration	Slope:0.904 Intercept: −0.018	Slope: 1.140 Intercept: −0.153	Slope: 0.442 Intercept: 0.000	Slope: −0.236 Intercept: 0.135
	Brier Score	0.069	0.070	0.070	−0.001
	Hosmer–Lemeshow *p*-value	0.545			

AF, atrial fibrillation; AUC, area under the curve; MVA, mitral valve area.

### 3.4 Diagnostic performance at optimal cut-offs

At their optimal Youden thresholds (Table 4), the D-dimer–only model achieved perfect sensitivity (100.0%, 95% CI: 73.5–100.0%) and a specificity of 71.4%, yielding a negative predictive value (NPV) of 100.0% (95% CI: 91.2–100.0%) and an overall accuracy of 0.765. Notably, applying the conventional 500 ng/mL threshold to our cohort yielded a markedly lower sensitivity of 75.0% (95% CI: 42.8–94.5%). This conventional cutoff failed to detect a quarter of the active LAT cases, thereby compromising clinical safety for rule-out purposes. Furthermore, the composite D-dimer+AF model exactly reproduced the performance metrics of the D-dimer-only model, reaffirming the absence of added discriminative contribution from AF. In contrast, the D-dimer+MVA model showed a different diagnostic balance, reaching a lower sensitivity of 75.0% but perfect specificity (100.0%), with the highest accuracy among all models (0.956) and the best positive predictive value (PPV) (100.0%).

**Table 4:** Diagnostic performance of D-dimer–based models at optimal youden probability thresholds for predicting LAT in rheumatic SMS patients.

Model	Threshold/Cutoff	Sensitivity (95% CI)	Specificity (95% CI)	PPV (95% CI)	NPV (95% CI)	Accuracy	Balanced accuracy
D-dimer–only	0.064^a^	1.000 (0.735–1.000)	0.714 (0.578–0.827)	0.429 (0.245–0.628)	1.000 (0.912–1.000)	0.765	0.857
D-dimer+AF	0.056	1.000 (0.735–1.000)	0.714 (0.578–0.827)	0.429 (0.245–0.628)	1.000 (0.912–1.000)	0.765	0.857
D-dimer+MVA	0.615	0.750 (0.428–0.945)	1.000 (0.936–1.000)	1.000 (0.664–1.000)	0.949 (0.859–0.989)	0.956	0.875
Conventional D-dimer	500 ng/mL	0.750 (0.428–0.945)	0.786 (0.656–0.884)	0.429 (0.218–0.660)	0.936 (0.825–0.987)	0.779	0.768

AF, atrial fibrillation; CI, confidence interval; MVA, mitral valve area; NPV, negative predictive value; PPV, positive predictive value. Balanced accuracy, (sensitivity+specificity)/2. Identical performance between D-dimer–only and D-dimer+AF models indicates no additional discriminative contribution from AF at the optimal Youden threshold.

a The predicted probability threshold of 0.064 in the D-dimer-only model corresponds to the optimal laboratory cutoff of 360.6 ng/mL.

## 4 Discussion

This study enhances current diagnostic evidence on D-dimer by shifting from conventional accuracy-based evaluation to a validated multimethod predictive modeling framework. Using Firth's penalized logistic regression with comprehensive calibration and bootstrap validation, D-dimer was independently associated with LAT and demonstrated strong predictive performance in the final model. Low D-dimer concentrations demonstrated excellent sensitivity and NPV at the optimal threshold (360.6 ng/mL). Conversely, although elevated levels suggest active thrombogenesis, they lack the specificity required to confirm LAT, thereby supporting the clinical utility of D-dimer primarily as a rule-out biomarker. Composite models incorporating AF or MVA achieved comparable discrimination but showed little evidence of meaningful incremental predictive value beyond D-dimer alone. Overall, these findings highlight D-dimer as a simple, reproducible, and cost-effective tool that may assist in identifying patients who could be considered for TEE deferral in rheumatic SMS.

Beyond the excellent discrimination of the D-dimer-only model, the practical clinical utility of any predictive tool heavily relies on its calibration. Our models demonstrated excellent calibration-in-the-large, with calibration intercepts closely approaching zero. This suggests that the average predicted risk of LAT was broadly consistent with the observed prevalence within the cohort. In the context of clinical decision-making, this strong calibration is paramount; it ensures that the predicted probabilities are highly reliable, thereby providing greater confidence in the potential clinical usefulness of the model for risk estimation and resource allocation decisions.

In rheumatic SMS, the three components of Virchow's triad, endothelial injury, stasis, and hypercoagulability (20), act synergistically to promote intra-atrial thrombus formation, even in patients who remain in sinus rhythm (21, 22). In our cohort, LAT was detected in 17.6% of patients, and SEC in 23.5%, reflecting marked atrial stasis and a prothrombotic milieu. This prevalence aligns closely with rates reported in prior research on RMS (11%–16%) (4, 11, 23) and inadequately anticoagulated AF populations (21%) (24). It is important to note that the presence of treatment-naïve patients with valvular AF in our cohort reflects the profound socioeconomic and healthcare access barriers in Yemen. Many of these patients present late from remote rural areas, where financial constraints and a lack of primary care infrastructure contribute to severe non-compliance or a complete lack of prior anticoagulant prescription. By focusing exclusively on these newly detected, untreated patients, our analysis was designed to evaluate the association between D-dimer levels and LAT in the absence of potential confounding from anticoagulant therapy, free from the pharmacological suppression of fibrin turnover caused by anticoagulant therapy (25).

Building on this methodological clarity, our analysis identified a D-dimer cutoff of 360.6 ng/mL as the optimal threshold. While our predictive model demonstrated 100% sensitivity and negative predictive value at this threshold within the study cohort, these diagnostic metrics must be interpreted with strict scientific caution rather than absolute clinical certainty. Furthermore, the external validity of these findings must be contextualized within the specific clinical characteristics of our cohort—primarily relatively young, treatment-naïve patients with advanced rheumatic disease. This profile contrasts sharply with populations in high-income regions, where advanced age naturally elevates baseline D-dimer levels (10) and widespread anticoagulant use suppresses fibrin turnover. Consequently, while the identified threshold appeared to perform better than the conventional 500 ng/mL cutoff within our cohort (which missed several LAT cases in our cohort), its performance may vary in different demographic or well-anticoagulated populations, necessitating robust external validation prior to broader implementation.

Regarding the clinical variables, we employed a hybrid approach to predictor selection. While AF and severe MVA are pathophysiologically linked to stasis and thrombus formation (26–28), our composite models indicate that their incremental predictive value is highly limited when D-dimer is already known. In this treatment-naïve cohort, D-dimer alone may reflect aspects of the thrombotic milieu associated with both rhythm and structural abnormalities, resulting in limited additional predictive contribution from AF and MVA for LAT rule-out within this cohort (12, 29–31). Nevertheless, these findings should not be interpreted as diminishing the clinical importance of AF or severe mitral valve narrowing. While D-dimer alone showed strong rule-out performance in this cohort, patients with elevated D-dimer levels may still benefit from additional clinical risk stratification. In this context, the coexistence of AF or severe anatomical obstruction may be associated with a higher post-test probability of LAT, helping to identify individuals who may warrant prioritization of TEE or closer evaluation. To properly contextualize these findings within a broader cardiovascular diagnostic paradigm, it is important to note that the utility of D-dimer as a highly sensitive rule-out biomarker extends well beyond VTE. For instance, D-dimer has demonstrated a robust exclusionary role in other severe cardiovascular emergencies, such as acute aortic dissection, where its high NPV has been widely used to support exclusion of the disease in appropriately selected patients (32). Our findings in RMS are broadly consistent with this established clinical paradigm.

Ultimately, it is essential to distinguish between the clinical application of D-dimer as a rule-out versus a rule-in biomarker. Because elevated D-dimer levels are inherently non-specific and may reflect a generalized prothrombotic or fibrinolytic state rather than the definite presence of a macroscopic thrombus, D-dimer alone is not suitable for confirming LAT. In contrast, a D-dimer level below the identified threshold was associated with an extremely low likelihood of LAT in our cohort. Given its observed 100% sensitivity and negative predictive value in this cohort, D-dimer showed considerable promise as a rule-out biomarker in treatment-naïve patients with rheumatic SMS. If validated in larger prospective multicenter studies, this strategy could potentially reduce reliance on routine TEE and improve patient triage efficiency in resource-limited settings.

### 4.1 Limitations

Several limitations of this study should be acknowledged. First, as a single-center study focusing on a highly specific and strict clinical phenotype, the sample size was inherently modest, with a limited number of LAT events. This strict selection resulted in wide CIs for our diagnostic accuracy metrics, introducing statistical uncertainty. Second, the study population represented relatively young, treatment-naïve patients with advanced RMS. Although the exclusion of patients receiving anticoagulant therapy minimized pharmacologic confounding, it may have introduced selection bias. Consequently, the observed findings may not be directly generalizable to older patients or anticoagulated populations. Third, despite excluding patients with overt systemic inflammation, infection, malignancy, renal failure, recent surgery, trauma, or thrombotic events, unrecognized subclinical variations in these conditions may still have influenced circulating D-dimer levels. Fourth, there are inherent constraints related to the biomarker measurements. Levels were obtained at a single time point, precluding the assessment of temporal variability or longitudinal changes. Additionally, concentrations were determined using a specific automated latex-enhanced immunoturbidimetric assay reported in FEU. Given the globally recognized lack of standardization across commercial D-dimer platforms, the proposed numerical cutoff may require local validation and calibration prior to application in other institutions. Fifth, continuous predictors, specifically D-dimer and MVA, were entered as linear terms on the logit scale. Although non-linear relationships cannot be excluded, the limited sample size and low event count constrained the use of more flexible approaches such as restricted cubic splines, which would have consumed additional degrees of freedom and increased the risk of model overfitting and unstable parameter estimates. Finally, while bootstrap internal validation demonstrated minimal optimism in model discrimination and calibration, it cannot completely eliminate the residual risk of overfitting given the limited number of outcome events (*n* = 12). Internal validation assesses reproducibility within the source population but does not guarantee transportability to external populations. Consequently, some degree of performance attenuation is expected when the model is applied to independent cohorts with different demographic characteristics, disease severity, comorbidity profiles, inflammatory burdens, or anticoagulation practices. This consideration may be particularly relevant in older, non-Yemeni populations and in healthcare systems with substantially different patterns of rheumatic heart disease presentation. Therefore, external validation in larger, multicenter cohorts using standardized imaging and laboratory protocols is warranted to confirm these findings and establish the broader applicability of the proposed D-dimer threshold.

## 6 Conclusions

Our findings suggest that D-dimer may represent a promising standalone biomarker for ruling out LAT in relatively young, treatment-naïve patients with RMS. At the identified threshold of 360.6 ng/mL, D-dimer demonstrated 100% sensitivity and negative predictive value within this cohort and appeared to perform better than the conventional 500 ng/mL cutoff, which failed to identify several LAT cases. Furthermore, incorporating clinical or echocardiographic variables into composite models provided little evidence of meaningful incremental rule-out value, suggesting that D-dimer alone retained most of the predictive information captured by the evaluated variables. While these findings indicate potential utility for patient triage and reducing reliance on routine TEE in resource-limited settings, they should be interpreted cautiously given the limited sample size and require prospective multicenter validation before routine clinical implementation.

## Data Availability

The raw data supporting the conclusions of this article will be made available by the authors, without undue reservation.
